# An Assessment of the Epidemiology and Herd-Level Impact of the Fractured Humerus Epidemic in New Zealand Dairy Cattle, 2007–2015: Results from Four Studies

**DOI:** 10.3390/ani14030524

**Published:** 2024-02-05

**Authors:** Jaimie C. Hunnam, Kevin Lawrence, Zul Bahar A. Rashid, Ben Hitchcock, Scott McDougall, Alvaro Wehrle-Martinez, Jenny F. Weston

**Affiliations:** 1 Ausvet, 34 Thynne Street, Bruce, ACT 2617, Australia; jaimie.hunnam@ausvet.com.au; 2 School of Veterinary Science, Massey University, Private Bag 11-222, Palmerston North 4442, New Zealand; zulbahar75@gmail.com (Z.B.A.R.); a.wehrlemartinez@massey.ac.nz (A.W.-M.); j.f.weston@massey.ac.nz (J.F.W.); 3 Cognosco, Anexa Veterinary Services, P.O. Box 21, Morrinsville 3340, New Zealand; ben.hitchcock@vetlife.co.nz (B.H.); smcdougall@anexafvc.co.nz (S.M.)

**Keywords:** fracture, dairy cattle, epidemiology, incidence, humerus, New Zealand

## Abstract

**Simple Summary:**

Spontaneous humeral fractures in first- and second-lactation dairy cows are recognized as a serious welfare problem in New Zealand. However, the condition is sporadic, which means that simple epidemiological descriptions and estimates of the extent of the problem are lacking. By combining the data from four independent studies using a multi-method approach, we found that their occurrence is more common than previously thought, with potentially up to 12% of farms and 4,620 dairy cattle affected in the 2013/2014 lactation season. Furthermore, the condition exclusively affects first- and second-lactation spring calving dairy cows, up to 4 months post-partum, and may affect multiple animals on the same farm, in the same season, and over multiple seasons, which are not always consecutive. We suggest that the evidence presented in this paper places an urgent requirement on the New Zealand dairy industry to undertake prompt action to understand the determinants of this epidemic.

**Abstract:**

A multi-method approach integrating data from four independent sources was used to describe some key features of the epidemiology and estimate the herd and within-herd incidence of fractured humeri in New Zealand dairy cattle for the period 2007–2015. The first dataset was from a national case series where cases of humeral fractures in dairy cattle were identified by veterinarians across New Zealand between the 2007/2008 and 2011/2012 lactation seasons. The second dataset was from a pet food company based in the Waikato region, which collated the number of casualty first- and second-lactation cows found to have a fractured humerus post-slaughter in the 2014/2015 lactation season, and the third dataset was a case series conducted by veterinarians employed in a Waikato veterinary business, also from the 2014/2015 lactation season. For the final dataset, 505 randomly selected New Zealand dairy farmers completed a phone survey on the incidence of non-responsive, non-weight-bearing forelimb lameness in first- and second-lactation cows in the 2014/2015 lactation season. Using the telephone survey results, the within-herd and herd-level incidence of cases for first- and second-lactation dairy animals was calculated. The national case series reported 149 cases of humeral fractures in 22 dairy herds; the pet food case series identified 61 cases from 41 farms; and the practice-based case series found 14 cases from 10 farms. Humeral fractures exclusively affected first- and second-lactation dairy cows and had a peak incidence between calving and early mating. The national telephone survey found that non-weight-bearing forelimb lameness requiring euthanasia of first- or second-lactation cows occurred in 11.7% of herds, with a mean within-herd incidence of 2.6% for first lactation cows and 2.8% for second-lactation cows for affected herds. These combined datasets demonstrate that humeral fractures in young, lactating dairy cattle are more common than previously suspected and that they occur nationally and over multiple years on some farms. Further work on this condition is urgently required in New Zealand to establish cost-effective management practices that will reduce unnecessary animal suffering and waste.

## 1. Introduction

In 2008, a case series of severe, non-weight-bearing forelimb lameness due to confirmed, spontaneous humeral fractures in first-lactation dairy cows in New Zealand was reported from a farm in the Manawatu region [1]. Subsequently, cases have been regularly reported from the Waikato, Bay of Plenty, Manawatu, Canterbury, Otago, and Southland regions to veterinarians at the School of Veterinary Science, Massey University (Palmerston North, New Zealand), from the regional veterinary laboratories (New Zealand Veterinary Pathology and Gribbles Veterinary Pathology), in Surveillance biosecurity magazine [2,3,4,5,6,7] and at veterinary congresses [8]. Surveillance is a quarterly magazine that reports on the Ministry for Primary Industry’s (MPI) biosecurity surveillance and the health status of New Zealand’s animal and plant populations (in terrestrial and aquatic environments).

Those reports on cases of humeral fractures show that on many properties, multiple animals were affected, and, anecdotally, the herd-level incidence appeared to be increasing [5,8]. Although copper deficiency was associated with humeral fractures in some cases, this was not a consistent finding, and more recent work has shown that this potential association is likely spurious [9]. Instead, histopathological examination of humeri from affected and non-affected first-lactation cows has led to the hypothesis that inadequate nutrition, resulting in insufficient deposition of bone during critical growth periods, leads to osteoporosis and spontaneous fracture [8,10,11].

**Figure 1:**
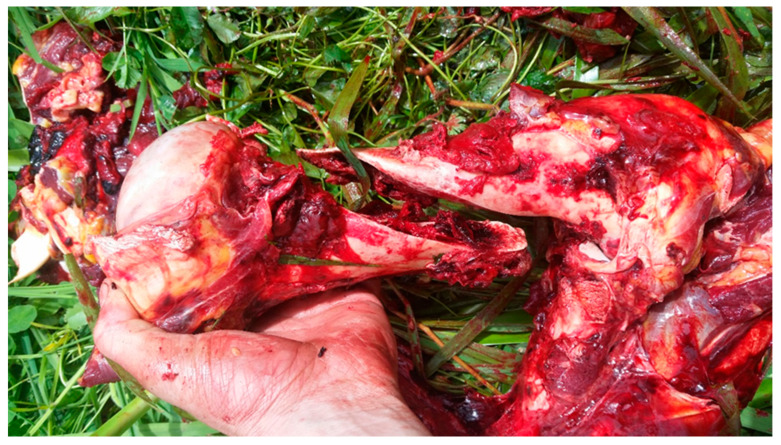
Typical spiral fracture of the mid-shaft of the humerus seen in affected animals (Photo D. Butler).

**Figure 2:**
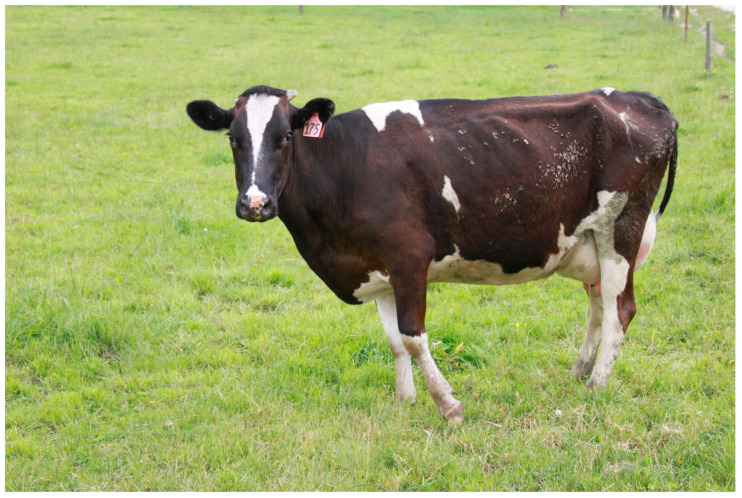
Characteristic “hanging leg” stance of cows affected by spontaneous humeral fracture (Photo D. Butler).

The fractures themselves are almost always a complete spiral fracture of the mid-shaft of the humerus (Figure 1), and affected cows present with a characteristic “hanging leg” stance, where the affected limb is elevated and hangs (Figure 2). Affected cattle also have a characteristic gait where the affected leg swings but rarely touches the ground (Videos S1 and S2, Supplementary Materials). Some farmers have reported hearing the fracture occur when the cows are being walked to the milking shed [2], although usually animals are just found injured in the paddock. Furthermore, the fact that, since 2008, only a limited number of cases of spontaneous humeral fractures have been reported outside New Zealand [12] supports the hypothesis that this condition may be unique to New Zealand and the New Zealand dairy system.

The New Zealand dairy system is mainly pasture-based and characterized by intensely seasonal calving, with over 95% of all dairy cows calving in the spring (July, August, and September). Replacement cows first enter the dairy herds at 2 years of age. Those spring-calving cows are then mated in October, November, and December. Milking on most farms occurs twice daily, although on some farms once-daily milking is preferred due to an associated reduction in farm costs, milk shed expenses, and the improvement of the mental health of farmers and staff [13]. Despite the total number of dairy farms in New Zealand declining, dairy farms themselves have grown in both size and production in the early 21st century, increasing from an average herd size of 251 cows producing 310 kg of milk solids/cow in 2000/01 to an average herd size of 419 cows producing an average of 377 kg of milk solids/cow in the 2014/15 season [14,15]. In addition, compared to dairy systems abroad, the New Zealand dairy system uses very little off-pasture housing and very little concentrate feeding, with pasture as the main source of dry matter intake for all ages of dairy cattle [16].

Prior to 2008, reports of lameness originating from the upper forelimb and/or shoulder in young dairy cattle, particularly due to a fracture, were rarely reported in New Zealand or internationally. Of 99 cases of cattle (mean age = 5.6 months) admitted to the University of Liège Veterinary Clinic (Belgium) with a limb fracture between 2000 and 2003, only 3 had suffered a humeral fracture [17]. Similarly, Crawford and Fretz (1985) reported that fractures in cattle occurred with the highest frequency in the femur, with only 12 of 213 (5.6%) cases observed in the humerus [18].

Multi-method research is a sub-category of mixed-methods research in which different data collection techniques and their associated analyses are combined; however, in multi-method research, this is restricted to either the quantitative or qualitative paradigm [19]. Proponents of mixed-methods research suggest that bringing different types of data together can strengthen research by offering multiple perspectives that both increase validity and generalizability [20]. Furthermore, mixed methods can be concurrent, where all studies are carried out at the same time, or sequential, where studies occur one after the other and data from earlier studies inform the focus of later studies [20].

This paper describes a multi-method approach to integrating the results of three independent case series, one undertaken nationally, one undertaken at a regional level in the Waikato, and one undertaken at a practice level also in the Waikato region, and a national randomized phone survey. The combined results from these studies improve our understanding of a condition that, for the last 15 years, has caused significant animal suffering and financial loss to the New Zealand dairy industry.

The aims of this research were:

- To describe key epidemiological features of humeral fractures in young, adult dairy cows. Including animal age and breed, postpartum time to fracture, seasonality, and regional distribution.
- To determine the within-herd and herd-level incidence of non-responsive, non-weight-bearing forelimb lameness in first- and second-lactation dairy animals across New Zealand.

## 2. Materials and Methods

**Figure 3:**
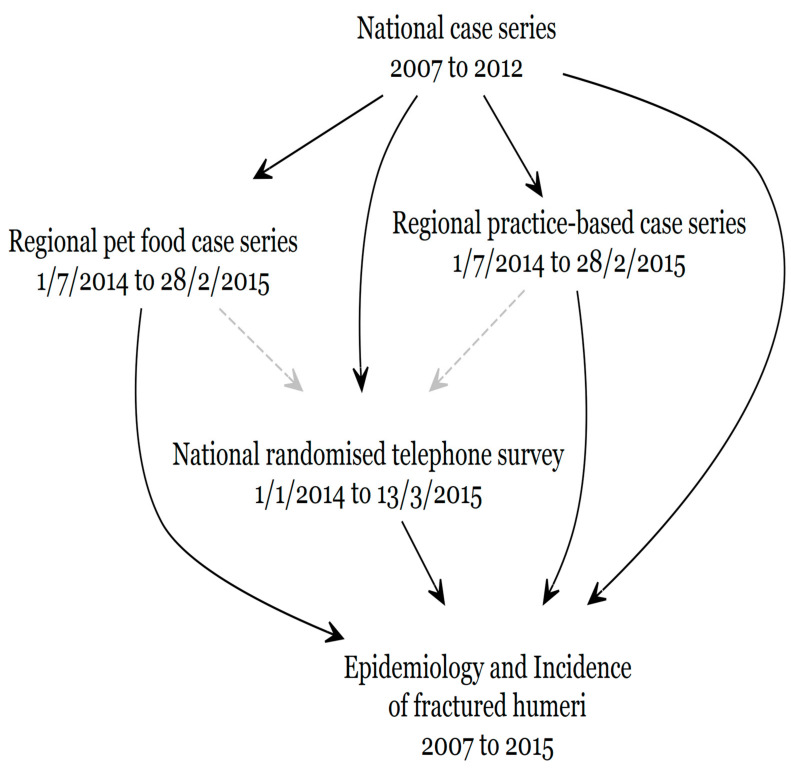
Causal diagram illustrating how the information from each of the four independent studies was integrated, the gray broken lines indicate only weak influence due to similar time periods.

This observational study integrated data from four independent quantitative studies: a national case series, a regional pet food case series in the Waikato region, a practice-based case series also in the Waikato region, and a national randomized telephone survey. Figure 3 illustrates how the four studies were amalgamated to reach conclusions on the epidemiology and incidence of humeral fractures in New Zealand dairy cows from 2007 to 2015.

### 2.1. National Case Series

In December 2011, approximately 500 New Zealand dairy veterinarians were contacted via newsletter and/or email to identify dairy herds among their clients where a spontaneous humeral fracture had been diagnosed within the previous 5 years. Owners/managers of affected herds completed a comprehensive questionnaire describing cases of acute, non-responsive, non-weight-bearing forelimb lameness in young dairy animals with no signs of external trauma. Herd-level information collected included the herd’s history, demographic information, and grazing history. Individual case information included a case description, reproductive and lactational status at the time of the fracture, and animal management [21]. Non-responders were recontacted at approximately 30-day intervals with a reminder letter and questionnaire.

### 2.2. Waikato Region Pet Food Case Series

A company based in the Waikato region of New Zealand that collected livestock for processing into pet food collated data on first- and second-lactation dairy cattle slaughtered between 1 July 2014 and 28 February 2015. This company purchased casualty stock for the manufacture of pet food only (i.e., typically those animals that were unfit for human consumption and/or were unfit for transport to an abattoir). First- and second-lactation cows were diagnosed with a humeral fracture post-slaughter when the musculature was removed from the affected bone during processing.

### 2.3. Waikato Region Veterinary Clinic Case Series

The third study was a case series conducted by veterinarians employed in a veterinary business (Anexa Veterinary Services, 25 Moorhouse Street, Morrinsville, New Zealand) that serviced approximately 800 dairy herds through fourteen clinics across the Waikato region. Case details were recorded after a veterinary diagnosis of any skeletal fracture in a dairy animal of any age-class between 1 July 2014 and 28 February 2015. Data collected included animal age, breed, calving date, fracture site, clinical signs, treatment/euthanasia plan, and likely cause of the fracture (e.g., trauma). Humeral fractures were diagnosed based on clinical signs, including non-weight-bearing lameness, palpable crepitus, and visible swelling over the fracture site.

### 2.4. National Telephone Survey

A national phone survey was undertaken with the aim of obtaining data on the incidence of non-responsive, non-weight-bearing forelimb lameness in first- and/or second-lactation dairy heifers from a minimum of 500 New Zealand dairy herds. Assuming a true herd-level incidence of 10% and a confidence level of 95% for the New Zealand dairy population of approximately 12,000 herds, the precision achieved with a sample size of 500 was 2.6%. Approximately 2500 herds from throughout New Zealand were randomly selected from the Client Relationship Management (CRM) database (DairyNZ) based on a predicted response rate of 20%, with the number of selected herds being proportional to the regional distribution of New Zealand dairy herds in the 2013/2014 lactation season (Table 1). Herds were randomly selected within each region.

Between 23 February 2015 and 13 March 2015, the primary contact for 1,278 herds was phoned by one of six experienced interviewers employed by a professional survey company (Versus Research Limited; King Street; Hamilton, New Zealand). Once the target sample size was achieved, no further herds were contacted. Eleven questions were asked concerning the history and demographics of the heifers and cows, including farmer knowledge of upper forelimb/shoulder lameness in those age-classes, monthly since 1 January 2014.

A first- or second-lactation cow was classified as having non-responsive, non-weight-bearing forelimb lameness if the farmer diagnosed lameness originating from the upper forelimb/shoulder and the animal was either euthanized as soon as possible (i.e., for home kill or pet food) or the lameness did not resolve after resting up to the end date of the survey (i.e., 13 March 2015). A first- or second-lactation cow with forelimb lameness that resolved after rest did not meet the case definition and was therefore excluded from further analysis.

### 2.5. Statistical Analyses

#### 2.5.1. National Case Series

Data were entered into Microsoft Excel (Microsoft; Redmond, WA, USA; 2010), and the herd owner/manager was contacted to clarify any missing values. Exploratory data analyses were performed in SAS (version 9.3; SAS Institute Inc., Cary, NC, USA) using the FREQ and UNIVARIATE procedures. The full data were tabulated by region and season, and descriptive statistics were presented by month of fracture, number of months postpartum when the fracture occurred, age of cow at fracture, and number of seasons that each farm experienced fractures.

#### 2.5.2. Waikato Region Pet Food Case Series

The overall proportion of first- and second-lactation dairy animals with a humeral fracture slaughtered by the pet food company relative to all dairy animals slaughtered was calculated, with 95% confidence intervals.

#### 2.5.3. Waikato Region Veterinary Clinic Case Series

Summary statistics by breed, age, and days postpartum were calculated.

#### 2.5.4. National Telephone Survey

Proportions and 95% confidence intervals were calculated using the exact binomial test. The incidence rates and 95% confidence intervals for non-responsive shoulder lameness for first- and second-lactation cow cows in affected herds were calculated by fitting a mixed effects negative binomial model to the data. The dependent variable was the number of heifers or cows with non-responsive, non-weight bearing-forelimb lameness; the total number of first- or second-lactation cows was included as an offset, and the region was fitted as a random intercept. The analysis was completed using the glmmTMB package [23] in R (version 4.1.1; R Core Team (2022). R: A language and environment for statistical computing. R Foundation for Statistical Computing, Vienna, Austria). 

There was no intention to amalgamate the data from the four studies; instead, the results from each study were compared and generalized where there was consensus.

## 3. Results

### 3.1. National Case Series

**Figure 4:**
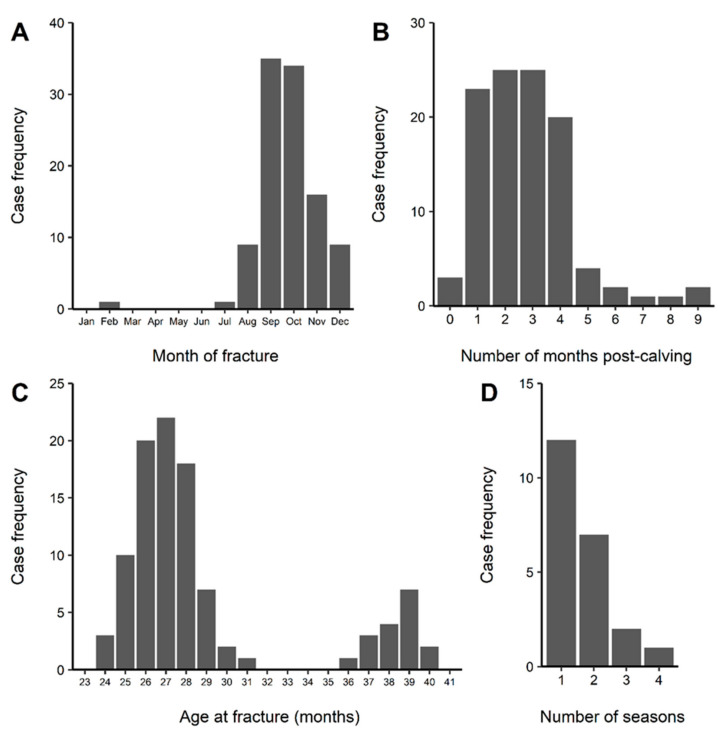
Frequency histograms of (**A**) month of fracture, (**B**) number of months post-calving when the fracture occurred, (**C**) age of bovine at fracture, and (**D**) number of seasons that farms had fractures for data collected in the national case series.

Owners/managers of 22 out of 30 identified herds completed a comprehensive questionnaire describing cases of sudden, non-responsive, non-weight-bearing forelimb lameness (i.e., suspected humeral fractures) in young dairy animals with no signs of external trauma. From these study herds, 149 cases of humeral fracture were reported between the 2007/08 and 2011/12 lactation seasons, with 58% physically examined and the humeral fracture confirmed by a veterinarian (Table 2). Study herds were in the following regions: Auckland (*n* = 1), Bay of Plenty (*n* = 1), Waikato (*n* = 14), Manawatu (*n* = 4), North Otago (*n* = 1), and Canterbury (*n* = 1). Ten study herds (45.5% (95% CI = 24.4–67.8%)) reported cases in more than one season, with one farm reporting cases over four successive seasons (Figure 4D). Individual farmers reported between 1 and 18 cases per season (mean 4.3 cases per season per farm), whereas on each affected farm, there were between 1 and 27 cases over the 5-year reporting period (mean 6.7 cases per farm).

Details were available for 115 individual cases, with 20% observed in Friesian, 21% in Jersey, and 59% in cross-bred animals. During the period of data collection (2007–2012), the proportion of cross-bred cows in the national herd increased from 31.6% to 40.8% [24,25]. Using the 2012 figure, i.e., the national proportion of cross-bred cows was 40.8%, the exact binomial test showed strong evidence that the proportion of cross-bred cows with humeral fractures was significantly different from the national prevalence of that breed (*p* = 0.0002). Cases were observed in all months between July and February, with peaks in September 35/105 (33.3%) and October 34/105 (32.4%) (Figure 4A). The age (in months) of 101 cases was provided, with all occurring between 24 and 40 months of age (Figure 4C). The plot is bimodal with two distinct age group peaks: 24–31 months (n = 84; mean = 26.8 months) and 36–40 months (n = 17; mean = 38.4 months). Overall, ninety-five cases (82.6%) occurred in first-lactation cows and 17 (14.8%) were cows in their second lactation, and although three first-lactation cows (2.6%) fractured in the final month of their first pregnancy, most cases, 93/106 (88%), occurred in the first-4 months post-partum. (Figure 4B). The affected limb was not recorded in all cases, but for those where that information was available, 37 (54%) occurred in the right limb, 31 (45%) in the left limb, and one animal fractured both forelimbs.

### 3.2. Waikato Region Pet Food Series

**Figure 5:**
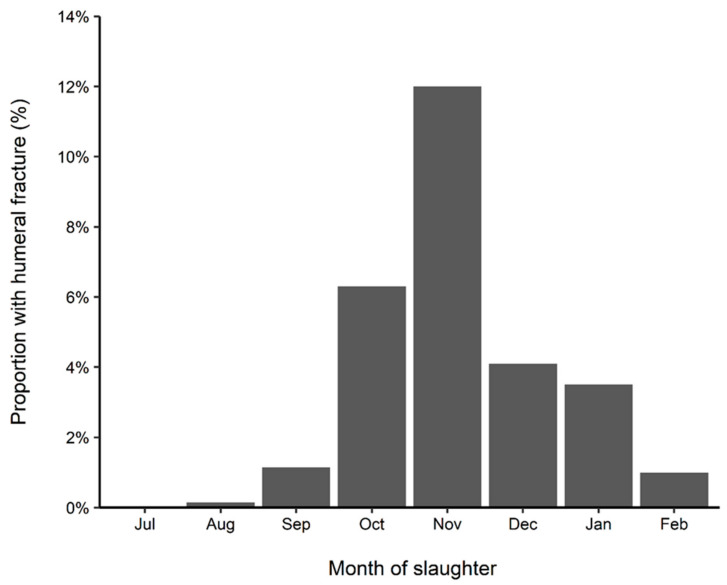
Proportion of the total dairy animals slaughtered by a pet food company in the Waikato region of New Zealand, per month, that were first- or second-lactation dairy animals with a humeral fracture between July 2014 and February 2015.

During the 2014/2015 lactation, a total of 61 first- or second-lactation dairy cows from 41 properties in the Waikato region had humeral fractures confirmed by pet food company employees. One animal was diagnosed on each of 39 properties, nine on one property, and 13 on the remaining property. Although cases were diagnosed every month during this study period, the relative frequency of cases peaked from late September 2014 to early December 2014. Between 1 July 2014 and 28 February 2015, the pet food company slaughtered 2,310 animals, with a minimum of 192 animals (December 2014) to a maximum of 496 animals (August 2014) per calendar month. The overall proportion of all slaughtered animals that were first- or second-lactation dairy animals with a humeral fracture was 61/2,310 (2.6% (95% CI = 2.0–3.4%)), with a peak in monthly incidence in November 2014 at 12.0% (Figure 5).

### 3.3. Waikato Region Veterinary Clinic Case Series

Ten Anexa Veterinary Services veterinarians diagnosed a total of 14 dairy animals of all ages with a humeral fracture between 1 July 2014 and 28 February 2015. All 14 animals were first-lactation cows and originated from 10 different properties (six properties, a single case each, and four properties, two cases each). Of the 14 first-lactation cows, 8/14 (57%) were Friesian, 2/14 (14%) were Jersey, and 4/14 (29%) were cross-bred. Ten (71%) animals were affected on the left and four (29%) on the right forelimb. The first-lactation cows were between one and 105 days postpartum at the date of diagnosis of humeral fracture (data only available for n = 7 animals). Three (30%) farmers had observed similar clinical signs in first-lactation cows in the previous (2013/14) lactation season.

### 3.4. National Phone Survey

Of the 1278 herds contacted to complete the phone survey, 542 farmers (42.4%) declined, 81 (6.3%) were no longer dairy farmers, sixteen (1.3%) were business associates but not the manager of the farm, and 79 contacted numbers (6.2%) were not in service. A further 55 farmers (4.3%) did not have primary responsibility for the dairy herd or their first-lactation cows calved in autumn or year-round. Despite this, approximately 20% of the pre-selected herds in each region completed a survey.

Surveys were completed by 505 farmers who had primary responsibility for a spring-calving dairy herd for a minimum of two years, including the current lactation season. Overall, one or more first- and/or second-lactation cow(s) was observed with non-responsive, non-weight-bearing forelimb lameness that required immediate euthanasia (home kill or pet food) or did not show improvement after rest in 59/505 herds (11.7% (95% CI = 9.0–14.8%)) between 1 January 2014 and 15 March 2015. Of those herds, 22/59 (37.3% (95% CI = 25.0–50.9%) had both first and second lactation cows affected, whereas 24/59 (40.7% (95% CI = 28.1–54.3%) and 13/59 herds (22.0% (95% CI = 12.3–34.7%) contained either only first- or second-lactation cow(s) affected, respectively. As illustrated in Table 3, affected first lactation cows were observed in herds located in all regions except Marlborough/Westland, whereas affected second lactation cows were not observed in study herds located in the Auckland/Northland, Bay of Plenty, or Marlborough/Westland regions.

#### 3.4.1. First-Lactation Cows

Seven respondents did not list the number of first-lactation cows that calved, and twenty-six farmers did not know whether any first-lactation cows had experienced lameness during this study period; both groups were removed prior to analysis. The mean and median number of first-lactation cows in the remaining study herds in 2014 were 105 and 85 animals, respectively (min 3; max 700). Overall, 46/472 (9.8% (95% CI = 7.2–12.8%)) farmers observed one or more first-lactation cows with non-responsive forelimb lameness in this study period. On those 46 farms, a total of 134 first-lactation cows were affected, representing 0.27% of the total number of first-lactation cows in this study population (n = 48,866). In affected herds, the mean incidence of first-lactation cows with non-responsive, non-weight-bearing forelimb lameness was 2.6% (95% CI = 2.0–3.4%; min 0.3%, max 11.2%).

#### 3.4.2. Second-Lactation Cows

Thirty respondents did not list the number of cows that commenced their second lactation in 2014, and twenty-three farmers did not know whether any cows had suffered forelimb lameness in 2014 or 2015. Both groups were removed prior to analysis. A further sixteen farms had no second-lactation cows, so they were also excluded. The mean and median number of second-lactation cows in the remaining study herds in 2014 were 101 and 70 animals, respectively (min 1, max 800). Overall, 34/436 (7.8% (95% CI = 5.5–10.7%) farmers observed one or more second lactation cows with non-responsive, non-weight-bearing forelimb lameness in this study period. On those 34 farms, a total of 115 second-lactation cows were affected, representing 0.23% of the total number of second-lactation cows in this study population (n = 47,997). In affected herds, the mean incidence of second-lactation cows with non-responsive, non-weight-bearing forelimb lameness was 2.8% (95% CI = 2.0–4.0%; min 0.14%, max 13.8%).

#### 3.4.3. Summary of Findings from the Four Studies 

Integrating the findings from the four studies, humeral fractures are widespread throughout New Zealand, and their occurrence is relatively common, with potentially up to 12% of dairy farms affected on an annual basis. The condition exclusively affects first- and second-lactation spring-calving cows, which are up to 4 months post-partum, and may affect multiple animals on the same farm, in the same season, and over multiple seasons. Furthermore, humeral fractures appear to be more common in crossbred cattle, and the on-farm incidence peaks over the September–November period.

## 4. Discussion

The use of a multi-method approach has confirmed that spontaneous humeral fracture was a serious and widespread problem in New Zealand between 2011 and 2015. Since then, the accumulated evidence shows that this problem has continued and possibly worsened [26,27,28,29,30,31].

Although there was good agreement between some elements of the four independent studies, this was not always the case. The national case series provided key epidemiology about the disease but clearly indicated that first-lactation cows were more at risk than second-lactation cows, whereas the national telephone survey showed that there was a slightly lower between-farm and slightly higher within-farm incidence of spontaneous humeral fracture in second-lactation cows compared to first-lactation cows. On the other hand, the practice-based case survey supported the national case series because all the affected cattle were first-lactation cows; however, there were only 14 cases from 10 farms. Unfortunately, this discrepancy could not be resolved by the pet food survey because the ages of cattle at slaughter were not accurately recorded. If we look more closely at the national telephone survey results, we find that although it is true that all case farms had first-lactation fractures, only around 59% of case farms had second-lactation fractures. Because the national case series only had 22 farms and the practice-based case series only had 10 farms, it is quite possible that these data sets had more farms with cases only in first lactation cows.

The national case series showed that the age distribution at fracture occurrence was bimodal, with peaks at 24–31 months and 36–40 months of age. Although there was no support from the pet food and practice case series, the national telephone survey again supported this finding with similar incidences of first- and second-lactation cow fractures on affected farms.

The national phone survey described in this paper indicates humeral fractures in first- and second-lactation cows occur in dairy herds located throughout New Zealand, with only Marlborough/Westland reporting no cases. This suggests that there are no regional risk factors such as soil type, pasture type, or weather associated with humeral fracture, but rather that the risk factors are ubiquitous throughout the New Zealand dairy industry. One potential risk factor is breed; in the last 40 years, there has been a rapid expansion in the population of crossbred cows in the New Zealand national dairy herd [32]. From the 1998/1999 season to the 2008/2009 season (when the first outbreak of humeral fractures was reported in New Zealand), there was a 15.9% increase in crossbred cows at the expense of Holstein-Friesian (14.2% less) [33,34]. Since then, this trend has continued, and as of the 2021–2022 season, crossbred cows are now 59.2% of the population of dairy cows in New Zealand [35]. The national case series study suggested that fractures were more common in crossbred dairy cows, and although this was not supported by the practice-based survey, a recent paper has identified that farms where crossbred cattle are the main dairy breed are 9.7 times more likely to have humeral fractures than farms with other dairy breeds [36]. Unfortunately, the breed of affected cattle was not asked about in the national telephone survey, so this did not support or contradict the findings of the national case series.

Within the national case series and Waikato pet food case series, humeral fractures appeared to follow a temporal pattern occurring in the first half of lactation, with a peak in late September and October. As the fractures coincided with the period of high calcium demand, due to most lactating cows reaching peak lactation in those months, transient osteoporosis associated with lactation may be the underlying cause. Furthermore, the peak occurrence of humeral fractures in dairy cows appears to coincide with the resumption of cyclicity post-calving, with the increased physical activity due to oestrus behavior likely contributing to fracture occurrence. Recent studies on humeral fractures in New Zealand have reported that the condition is associated with periods of inadequate feed quality, leading to decreased bone formation and increased abnormal bone resorption that severely affects bone quality and strength [9,10,11,37,38]. Some support for this hypothesis is provided by a study in yearling zebu steers where an experimental dietary protein deficiency applied over 100 days resulted in evidence of mild rib osteoporosis, indicating an association with phosphorus and calcium and decreased absorption [39].

The studies reported in this paper include the first cross-sectional, national evaluation of the incidence of non-responsive, non-weight-bearing forelimb lameness, focusing on humeral fractures, in first- and second-lactation dairy cows in New Zealand. The condition occurred on 9.7% and 7.4% of New Zealand dairy farms in the 2014/15 lactation season among first- and second-lactation cows, respectively, with a mean within-herd incidence of 2.6% and 2.8% in affected herds. Based on these results, the average economic loss associated with the euthanasia of dairy animals in the early stages of their productive lives due to non-responsive, non-weight bearing forelimb lameness appears to be significant for affected herds.

When designing the telephone survey, it was assumed that farmers would not fail to observe a cow with non-responsive, non-weight-bearing forelimb lameness, considering the clinical signs are immediate and visually striking. Nonetheless, it is acknowledged that those clinical signs may have been indicative of non-responsive lower forelimb disease rather than an upper forelimb fracture. However, as the focus of this paper was humeral fractures, and to increase the specificity of the diagnosis of this condition, the case definition for the phone survey was narrowed post-hoc to only include forelimb lameness that resulted in the euthanasia of the animal(s). We considered it unlikely that many cases of lameness resulting from a lower limb condition, such as a sole injury or white line disease, would result in euthanasia, whereas almost all cases of humeral fracture would require immediate or eventual euthanasia.

The authors have been unable to source published evidence of any other common causes of either upper or lower forelimb lameness in first- and second-lactation dairy animals of sufficient severity to meet the case definition for the telephone survey except for humeral fractures [40,41]. Therefore, although the dependence on farmer diagnosis within the phone survey means that the incidence figures calculated cannot be stated definitively to be due to humeral fracture, the authors conclude that this is likely.

Conversely, recall bias or the inability of an individual farmer to accurately recall all cases of humeral fracture in their first- and second-lactation cows may have resulted in an underestimation of the true number of dairy animals affected by this condition in the 2014/2015 lactation season. However, as the survey was conducted in the same lactation season as the cases, respondents were limited to those with primary responsibility for the dairy herd, and the condition of interest is visually striking, so the recall bias was expected to be minimal.

Under Section 138 of New Zealand’s Animal Welfare Act 1999 an animal should be destroyed if reasonable treatment will not be sufficient to expect a response or if the animal will suffer unreasonable or unnecessary pain or distress [42]. Although anecdotally, there have been reports of some heifers and cows with humeral fractures recovering after resting (<5%), the welfare costs for any animal with a fractured humerus are so great that all affected animals should, and mostly are, destroyed soon after diagnosis. The fact that some rested animals may recover could mean that the incidence of forelimb lameness in first- and second-lactation cows, as calculated from the national telephone survey, may have been a slight underestimate.

Aside from the animal welfare costs of this condition, there are significant financial losses for individual farmers and the dairy industry. There were 11,927 dairy herds in New Zealand in the 2013/2014 lactation season [21]. Assuming a median first- and second-lactation cow mob size of 85 and 70 animals, respectively, there were approximately 1.01 million first-lactation cows and 835,000 second-lactation cows present in that season. Based on the national survey data, if an estimated 11.7% of herds contained one or more first-lactation cows and/or second-lactation cows with non-responsive, non-weight-bearing forelimb lameness, then 1395 herds nationally were potentially affected. A national incidence of this condition in first-lactation cows of 0.27% would result in an estimated 2700 first-lactation cows affected nationally, whereas a national incidence in second-lactation cows of 0.23% would result in an estimated 1920 second-lactation cows affected nationally. Therefore, if the herd-level and within-herd incidence rates derived from the phone survey are representative of the dairy population, further dedicated research on the potential causes of this problem is urgently required.

A total of 4620 cattle experiencing a fractured humerus seems like an extraordinary and implausible number, and yet the pet food case series gave some support for this estimate. Collection by a pet food company is only one method of casualty animal disposal available to farmers; some cows that require immediate euthanasia are slaughtered on-farm for human consumption, some are shot and buried on farm, and/or are shot and collected by local hunt kennels. If we assume that the pet food company collected from 500 Waikato farms and only 50% of all animals with a fracture from those farms were sent to them, then potentially this is equivalent to 61 × 11,927/500 × 1/0.5 = 2910 first- and second-lactation cows with humeral fracture nationwide. A figure that is 63% of the national telephone survey estimate.

What is difficult to understand is the low farm incidence rate found by the practice-based case series, with only 10/800 (1.25%) client farms reporting cases, a fraction of the total affected farms if the national telephone survey is correct. It is possible that farmers were not keen to have their losses publicly known, and because many of the Anexa Veterinary Services farms were also serviced by the pet food company, there is clearly an issue with under-reporting of this condition by farmers. Underreporting has dogged much of the early research into this condition, including encouraging the New Zealand dairy industry to recognize the severity of the problem, and is one of the reasons why a multi-method approach was selected to gain more insight into this condition. Of interest is that 30% of the affected farms from the practice-based survey had cases the previous season. This is very similar to the findings from the national case series and shows that outbreaks of fractured humeri are not restricted to one season and tend to recur on some farms for up to four seasons, which are not always consecutive.

The number of cows experiencing a fracture on individual farms in a single season can be emotionally and financially distressing. With the national case series reporting two farms with 16 and 18 cases in the 2011/2012 season, the national phone survey had one farm report 30 combined first- and second-lactation fractures, and the pet food company collected 13 animals from just one property. What effect this has on the mental health of farmers is unknown, but the psychological effects on farmers that slaughter farm stock due to notifiable disease outbreaks are well recorded and are likely serious [43,44].

First-lactation cows (heifers) in New Zealand are commonly reared at geographically distant locations from the home farm, with often infrequent observation by the owner until they return as in-calf heifers at approximately 21 months of age [45]. The main goal for heifer management has typically been heifer liveweight at calving, as this has been identified as having a significant impact on milk production in the first lactation [46,47]. However, further evaluation of pre-weaned and weaned heifer nutrition and growth is warranted, focusing particularly on a detailed evaluation of significant risk factors for humeral fractures in heifers, as this may identify cost-effective management practices to limit the occurrence of this condition.

## 5. Conclusions

Humeral fractures in first- and second-lactation New Zealand dairy cattle are more common than previously suspected; they occur nationally and over multiple years on some farms. Further work on this catastrophic condition is urgently required in New Zealand to establish cost-effective management practices that will reduce unnecessary animal suffering and waste.

## Figures and Tables

**Table 1:** Regional distribution of New Zealand dairy herds that participated in a national phone survey assessing the incidence of humeral fractures in young dairy cows, relative to 2013/14 New Zealand dairy industry statistics.

Region	Study Herds		New Zealand ^1^
	n	%	%
Northland/Auckland	49	9.7	11.3
Waikato	144	28.5	29.6
Bay of Plenty	27	5.3	5.0
Taranaki	59	11.7	14.4
Lower North Island	71	14.1	13.9
Marlborough/Westland	26	5.1	5.1
Canterbury/Otago	78	15.4	12.8
Southland	51	10.1	7.9
**TOTAL**	**505**	**100.0**	**100.0**

^1^ Data from DairyNZ; LIC. New Zealand Dairy Statistics 2013–2014 [22].

**Table 2:** Number of cases of spontaneous humeral fracture observed in young New Zealand dairy cows in 22 herds over 5 lactation seasons (National case series).

Herd	Region	Lactation Season					TOTAL
		2007/2008	2008/2009	2009/2010	2010/2011	2011/2012	
**1**	Auckland	0	0	0	0	1	1
**2**	Bay of Plenty	0	0	4	0	0	4
**3**	Waikato	0	0	0	0	2	2
**4**	Waikato	0	0	0	0	2	2
**5**	Waikato	0	1	4	0	0	5
**6**	Waikato	0	0	0	0	2	2
**7**	Waikato	0	0	0	0	1	1
**8**	Waikato	0	3	2	3	5	13
**9**	Waikato	0	0	4	7	16	27
**10**	Waikato	0	0	1	0	1	2
**11**	Waikato	0	0	0	1	4	5
**12**	Waikato	0	0	0	0	18	18
**13**	Waikato	0	0	8	1	0	9
**14**	Waikato	0	0	2	8	2	12
**15**	Waikato	0	0	7	0	6	13
**16**	Waikato	0	0	0	0	3	3
**17**	Manawatu	0	0	0	0	2	2
**18**	Manawatu	0	0	1	0	3	4
**19**	Manawatu	7	0	0	0	0	7
**20**	Manawatu	0	0	0	2	3	5
**21**	North Otago	0	0	0	0	2	2
**22**	Canterbury	0	0	0	0	10	10
**TOTAL**		7	4	33	22	83	149

**Table 3:** Number (%) of herds that had one or more first-lactation cows, second-lactation cows, or either age-class with a humeral fracture (“Case herds”) from 1 January 2014 to 15 March 2015 based on a national phone survey of 505 New Zealand dairy herds, by region.

Region	Study Herds											
	First-Lactation Cows				Second-Lactation Cows				Either Age-Class			
	Case Herds		Non-Case Herds		Case Herds		Non-Case Herds		Case Herds		Non-Case Herds	
	n	%	n	%	n	%	n	%	n	%	n	%
Auckland/Northland	3	6.5	44	93.5	0	0.0	46	100.0	3	6.1	46	93.9
North Waikato	6	9.7	56	90.3	6	9.8	55	90.2	9	13.4	58	86.6
South Waikato	6	8.6	64	91.4	4	6.0	62	94.0	7	9.1	70	90.9
Bay of Plenty	1	4.0	24	96.0	0	0.0	23	100.0	1	3.7	26	96.3
Taranaki	1	1.8	54	98.2	1	2.0	49	98.0	2	3.4	57	96.6
Lower North Island	6	8.6	64	91.4	7	10.9	57	89.1	9	12.7	62	87.3
Marlborough/Westland	0	0.0	22	100.0	0	0.0	25	100.0	0	0.0	26	100
Canterbury/Otago	15	20.3	59	79.7	10	15.6	54	84.4	16	20.5	62	79.5
Southland	8	17.0	39	83.0	6	16.3	31	83.7	12	23.5	39	76.5
**TOTAL**	**46**	**9.8**	**426**	**90.2**	**34**	**7.8**	**402**	**92.2**	**59**	**11.7**	**446**	**88.3**

## Data Availability

Data are unavailable due to privacy.

## Supplementary Materials

The following supporting information can be downloaded at: https://www.mdpi.com/article/10.3390/ani14030524/s1. Video S1: Heifer with fractured humerus walking. Video S2: Heifer with a fractured humerus walking.
